# Crystal Structure of Cruxrhodopsin-3 from *Haloarcula vallismortis*

**DOI:** 10.1371/journal.pone.0108362

**Published:** 2014-09-30

**Authors:** Siu Kit Chan, Tomomi Kitajima-Ihara, Ryudoh Fujii, Toshiaki Gotoh, Midori Murakami, Kunio Ihara, Tsutomu Kouyama

**Affiliations:** 1 Department of Physics, Graduate School of Science, Nagoya University, Nagoya, Japan; 2 Center for Gene Research, Nagoya University, Nagoya, Japan; 3 RIKEN Harima Institute/SPring-8, Mikazuki, Sayo, Hyogo, Japan; University of the Basque Country, Spain

## Abstract

Cruxrhodopsin-3 (cR3), a retinylidene protein found in the claret membrane of *Haloarcula vallismortis*, functions as a light-driven proton pump. In this study, the membrane fusion method was applied to crystallize cR3 into a crystal belonging to space group *P321*. Diffraction data at 2.1 Å resolution show that cR3 forms a trimeric assembly with bacterioruberin bound to the crevice between neighboring subunits. Although the structure of the proton-release pathway is conserved among proton-pumping archaeal rhodopsins, cR3 possesses the following peculiar structural features: 1) The DE loop is long enough to interact with a neighboring subunit, strengthening the trimeric assembly; 2) Three positive charges are distributed at the cytoplasmic end of helix F, affecting the higher order structure of cR3; 3) The cytoplasmic vicinity of retinal is more rigid in cR3 than in bacteriorhodopsin, affecting the early reaction step in the proton-pumping cycle; 4) the cytoplasmic part of helix E is greatly bent, influencing the proton uptake process. Meanwhile, it was observed that the photobleaching of retinal, which scarcely occurred in the membrane state, became significant when the trimeric assembly of cR3 was dissociated into monomers in the presence of an excess amount of detergent. On the basis of these observations, we discuss structural factors affecting the photostabilities of ion-pumping rhodopsins.

## Introduction

Since the light-driven chloride ion pump halorhodopsin from *Natromonas pharaonis* (*p*HR) was discovered to have the ability to silence the electrical activity of neurons [1], a number of ion-pumping rhodopsins have proved useful for optical control of the neuron activity [2], [3]. Among them, the light-driven proton pump archaerhodopsin-3 (Arch) was reported to be most powerful in optically silencing neurons [4]. Meanwhile most microbial rhodopsins expressed in neural cells are rapidly inactivated under illumination. Although light-induced inactivation is less significant in Arch and its homologs, little is known about what structural factor determines the protein stability under illumination. To date, several proton-pumping rhodopsins [5]–[14] and two anion-pumping rhodopsins [15], [16] have been crystallized. Structural comparison of these proteins enabled the discussion about a common structural motif that is relevant to the ion-pumping activity [17]. However, it is still difficult to elucidate which structural factors affect the protein stability of ion-pumping rhodopsins in neuron cells. Against this background, it is important to accumulate more structural data of microbial rhodopsins.

**Figure 1:**
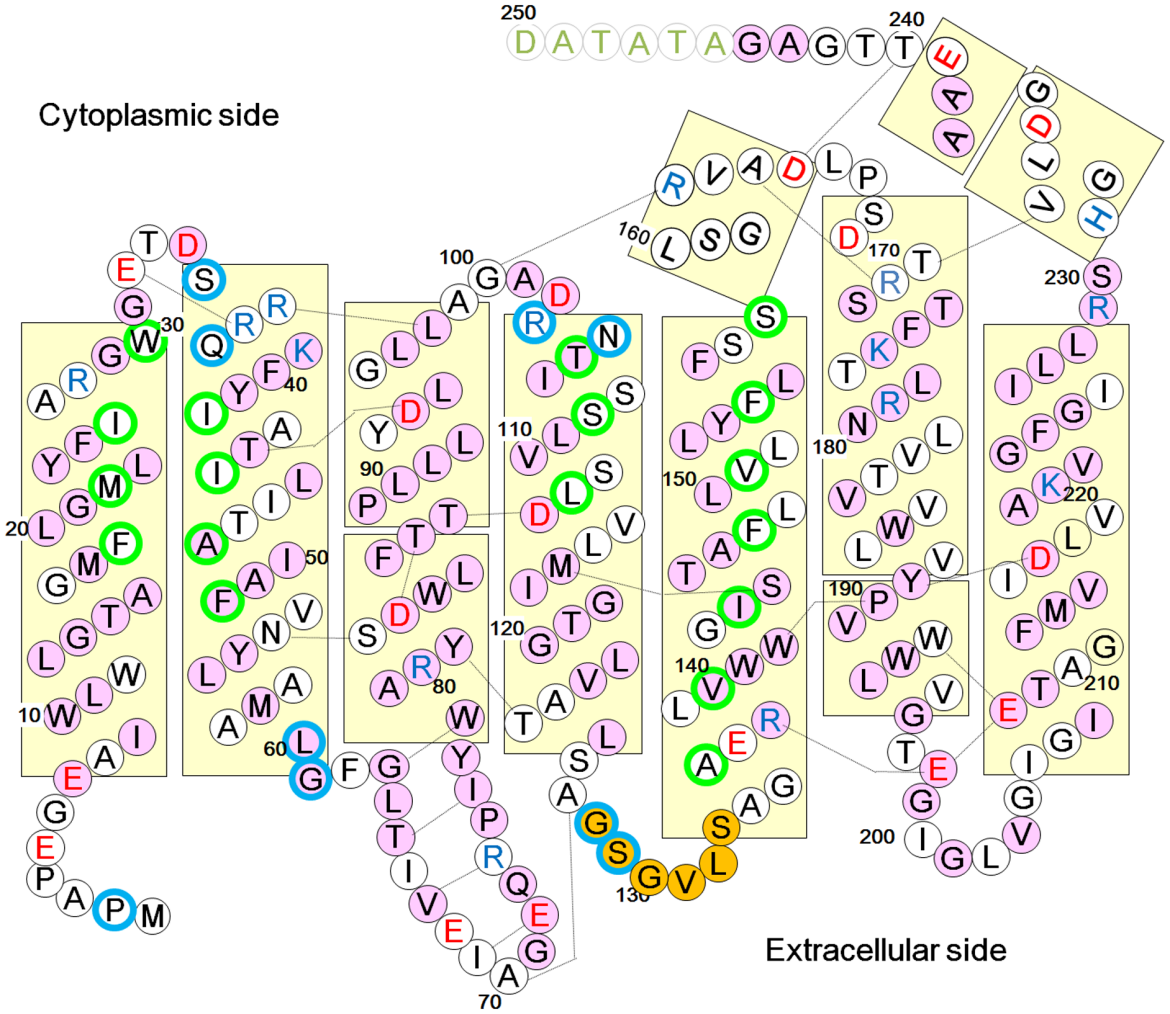
Schematic diagram of the topology of cR3 showing helices as rectangles. Residues contacting bacterioruberin and residues participating in intra-trimer protein–protein interactions are framed with green and blue circles, respectively. Pink circles represent residues conserved between cR3 and bR, whereas orange circles show inserted residues found only in cR3. Residues excluded from the structural model of cR3 are shown by grey letters.

Cruxrhodopsin (cR) is a member of the archaeal rhodopsin family and it functions as a light-driven proton pump. The presence of cruxrhodopsin-1 and -2 in *Haloarcula argentinensis* and *Haloarcula mukohataei*, respectively, was first reported by Mukohata and colleagues [18], [19]. A homologous protein, cruxrhodopsin-3 (cR3), was isolated from *Haloarcula vallismortis* [20]. Sequence identity among these homologs is high (>90%) [21]. But the amino acid sequences of all members of the cR family are distinct from those of the achaeal proton-pumping rhodopsins with known structure (i.e., sequence identities with bacteriorhodopsin (bR) are 48–54%) (Fig.1). Since cR works as a powerful neuron silencer [3], its structural information would provide a clue in engineering a novel neuron silencer with a more suitable structural and spectral property.

In the present study, we investigated spectroscopic and structural properties of cR3. This target protein was expressed in a bR-deficient mutant strain of *Halobacterium salinarum* (MPK409) and crystallized by the membrane fusion method [22]. Diffraction data showed that cR3 forms a trimeric assembly with bacterioruberin bound to the crevice between neighboring subunits, as previously observed in 3D crystals of archaerhodopsin-2 (aR2), deltarhodopsin-3 (dR3) and *pharaonis* halorhodopsin (*p*HR) [11], [12], [16]. Although the structure of the proton-release pathway is highly conserved among the proton-pumping rhodopsins, cR3 possesses the following peculiar structural features: i) The cytoplasmic vicinity of retinal is more rigid in cR3 than in bR; ii) The cytoplasmic end of helix E is greatly bent so that a large cavity is created between helices E and F; iii) The DE loop interacts with a neighboring subunit to strengthen the trimeric assembly; iv) Three positive charges are distributed along helix F. Meanwhile, it was observed that the decay kinetics of some photoreaction states of cR3 were significantly different from those reported for bR. For example, the decay rate of the K state was ten times slower in cR3 than in bR. It was also shown that in the solubilized state cR3 exhibited a much higher photostability than observed for bR. By comparing the structural and absorption kinetics data of cR3 with those of other ion-pumping rhodopsins, we discuss structural factors affecting the photoreaction kinetics of rhodopsins.

## Results

### Spectroscopic properties of cR3

**Figure 2:**
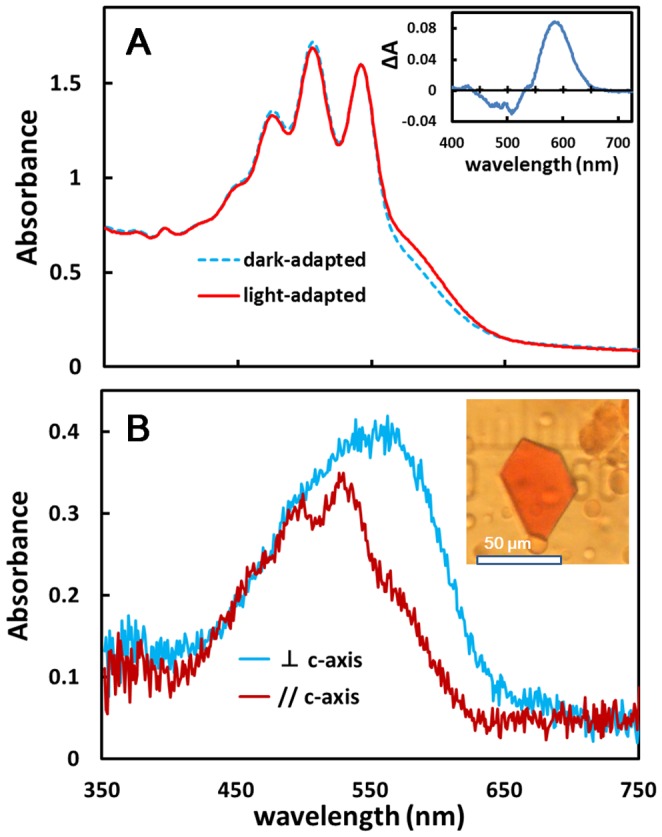
Spectroscopic properties of cR3-rich claret membrane and the *P321* crystal of cR3. *A*) Absorption spectra of dark- and light-adapted states of cR3 in claret membrane suspension at pH 7. Insert: Difference spectrum associated with the light adaptation of cR3. B) Absorption spectra of the *P321* crystal of cR3 at pH 4. The two spectra were recorded when the polarization plane of the measuring light was parallel (brawn) and perpendicular (cyan) to the crystal c-axis. Insert: Photograph of the *P321* crystal of cR3.

When the cells of *H. salinarum* that were transformed with the cop3 gene were repeatedly washed with distilled water, claret membrane vesicles containing cR3 as the major protein were isolated (Fig. S1). The absorption spectrum of purified claret membrane has three significant peaks at 475, 505, and 541 nm, which are attributable to the vibronic bands of bacterioruberin (Fig. 2A). The visible absorption band of retinal was recognized as a shoulder at the longer wavelength. A high content of bacterioruberin in claret membrane made it difficult to analyze the spectroscopic property of retinal in cR3. Meanwhile, a limited amount of bacterioruberin was incorporated into a trigonal crystal of cR3 (Fig. 2B). From the two absorption spectra of the light-adapted crystal that were measured with polarized light, it was shown that the absorption dipole moment of retinal is tilted largely from the c axis of the crystal, whereas the absorbance of bacterioruberin is significant only when the polarization plane of the measuring light is in parallel to the c axis. It was estimated that the retinal in cR3 has a broad absorption band with an absorption peak at ∼560 nm.

### Photoreaction of cR3

The difference spectrum associated with the light adaptation of cR3 exhibited a positive peak at 585 nm and a negative peak at 500 nm (Fig. 2A). Since this difference spectrum is similar to the corresponding spectrum of bR [23], it is suggested that the dark/light adaptation (i.e., thermal and light-initiated interconversion between the 13-*cis* and the all-*trans* retinal isomers) of cR3 takes place in a similar fashion as reported for bR.

**Figure 3:**
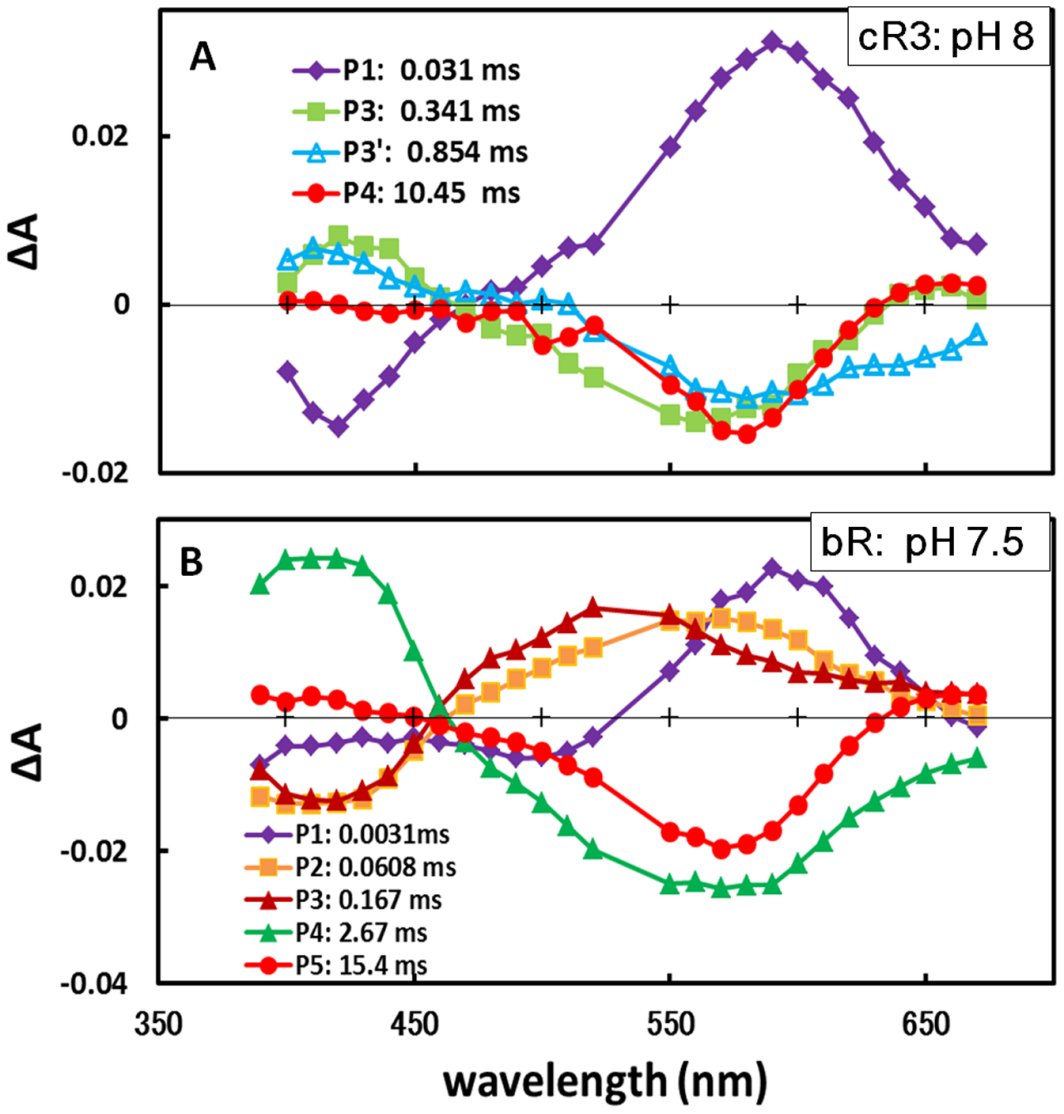
Photoreaction kinetis of cR3 and bR. A) Photoreaction kinetics of cR3 in the claret membrane of *Haloarcula vallismortis* suspended in 1 *M* KCl and 0.05 *M* HEPES at pH 8 and at 24°C. Flash-induced absorption changes measured at various wavelengths were fitted with four exponential components; in this panel, the amplitude of each component is plotted against the wavelength of the measuring light. B) Photoreaction kinetics of bR in the purple membrane of *Halobacterium salinarum* suspended in 1 *M* KCl and 0.05 *M* HEPES at pH 7.5 and at 24°C [49].

It has been shown from absorption kinetics data of the purple membrane of *H. salinarum* that the photoreaction cycle of the trans isomer of bR (bR_570_) is described by the reaction scheme: K → L → L/M → M → N → O → bR_570_, where the L/M state represents a rapid, dynamic equilibrium between L (L_2_) and M (M_1_) [24], [25]. It might be expected that the proton-pumping cycle of cR-3 is described by the same reaction scheme. In fact, the formation/decay rates of the individual reaction states of cR3 were considerably different from those observed for bR. Fig. 3A shows the absorption changes observed when cR3 in a membrane suspension was excited with light pulses at 532 nm. In the investigated time region, the absorption kinetics of cR3 were fitted with four exponential components (Fig. S2). At pH 8, the difference spectrum associated with the P_1_ component exhibited a positive peak at 590 nm and a negative peak at 410 nm, suggesting that the K state decayed into the M state. It is noteworthy that the K state of cR3 decays very slowly; i.e., its lifetime (∼30 µs at 24°C) is much slower than the lifetime (∼3 µs) of the K state of bR. The P_2_ component, which is characterized by the difference spectrum with a positive peak at 410 nm and a negative peak at 560 nm, is attributable to the transition from the M state to the N state. For the slower component (the P_3_ component), a negative peak was seen at 650 nm. This peak may be explained by supposing that the lifetime of N becomes much longer than that of the O state; in this case, the negative peak at 650 nm is interpreted to reflect the decay process of O. Indeed, the difference spectrum associated with the P_4_ component (τ = 10.5 ms) is nearly identical to the difference spectrum between bR_570_ and its N state [26].

On the basis of light-induced absorption changes observed under various solvent conditions, we propose that the photocycle of the trans isomer of cR3 is described by the following scheme: K → L/M → M → N → O → cR3 (Fig. S2). The L state, which occurs between the K state and the L/M state in the photocycle of bR, is not detected for kinetic reason. The L/M state becomes undetectable at high pH, where its decay rate is likely to be faster than the decay rate of the K state.

### Crystal packing

**Figure 4:**
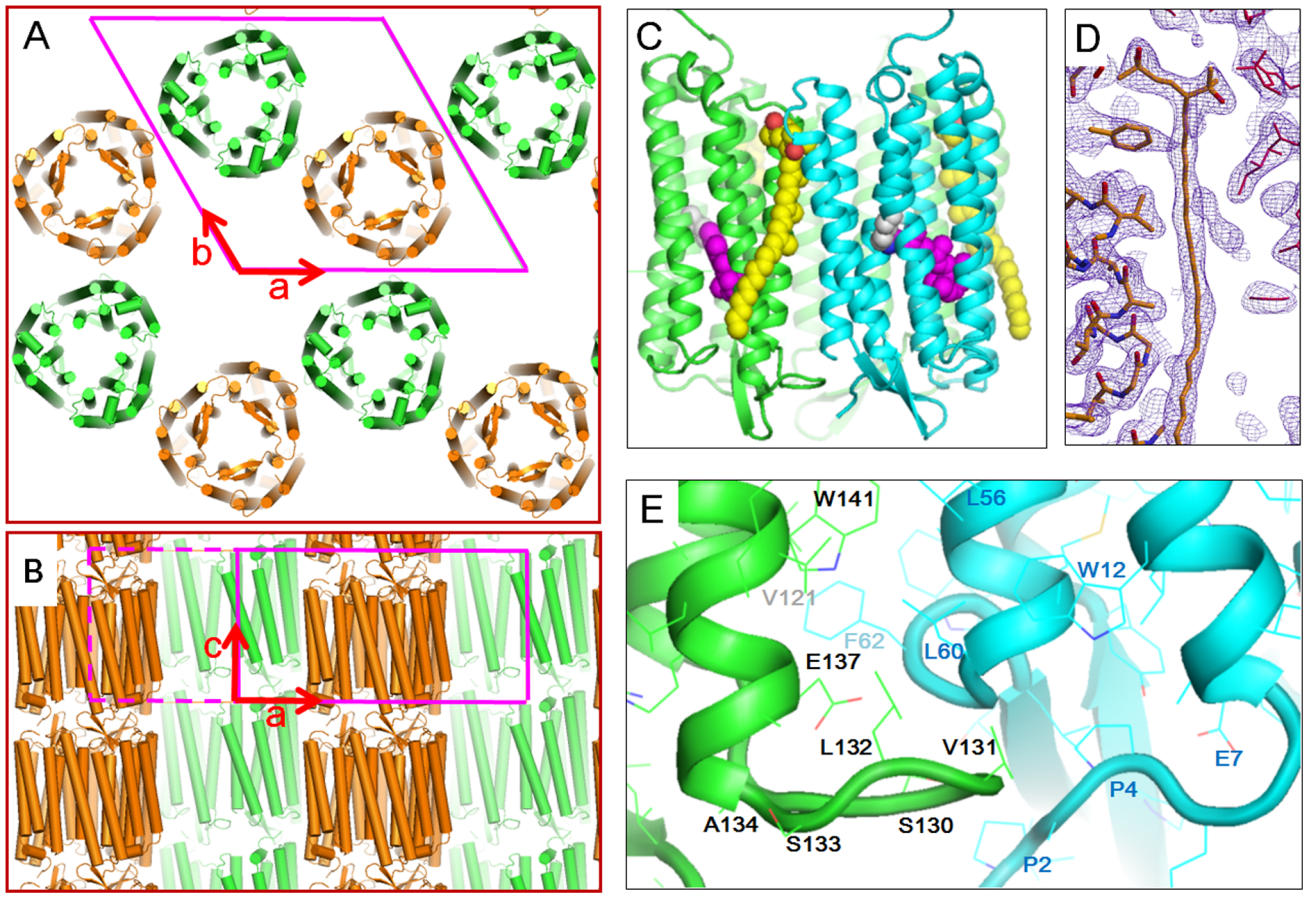
Crystal packing of cR3 in the *P321* crystal. A, B) the crystal packing at pH 5, viewed along the c-axis (a) and the a*-axis (**b**). C) The trimeric structure of cR3 in complex with bacterioruberin (yellow). D) 2*F_0_-F_c_* map around bacterioruberin, contoured at 1 σ. E) The DE loop in one subunit (green) extending towards a neighboring subunit (cyan).

Figure 4 shows the protein packing in the *P321* crystal of cR3. The side view of the unit cell shows that the *P321* crystal is composed of membranous layers. In each membranous layer, cR3 trimers are arranged on a honeycomb lattice such that neighboring trimers have opposite orientations. Along the c-axis, the membranous layers are piled up straightly. This crystal packing is similar to that seen in the *P321* crystal of aR2 [10]. But the cell dimension of the cR3 crystal (*a* = *b* = 106.2 Å and *c* = 55.2 Å) is noticeably larger than that of the aR2 crystal (*a* = *b* = 98.2 Å and *c* = 56.2 Å). The problem of crystal twinning, which was reported to be serious in the crystallization of aR2, was overcome when the *P321* crystal of cR3 was grown at pH 4.

The structural model of cR3 at the highest resolution was constructed using diffraction data from the crystal soaked at pH 5, though essentially the same crystal structure was observed at pH 4. At these low pH levels, Glu5 and Glu72 on the extracellular surface are close to Glu239 and Asp166, respectively, on the cytoplasmic surface of a neighboring subunit in a different membranous layer (Fig. S3). It would be expected that when these acidic residues are deprotonated at a higher pH, the electrostatic repulsive forces between them become strong enough to destroy the crystal structure. In fact, the cell dimension along the c axis increased significantly (by 5 Å) when the *P321* crystal was soaked in a post-crystallization solution at or above pH 6 (Table 1). It was observed that pH-induced conformational change and/or disorder in the BC loop and the C-terminal polypeptide was accompanied by a remarkable expansion of the inter-membrane space (Fig. S3). Nonetheless, the honeycomb structure of the cR3 trimers was little affected by the pH increase. It should be mentioned that no high-quality crystal of cR3 grew at neutral pH. So, it is possible that the crystal structure observed after the crystal soaking at neutral pH represents a quasi-stable state.

**Table 1:** Data collection and final refinement statistics.

Data collection				
Soaking solution	pH 4	pH 5	pH 6	pH 7
Resolution (Å)	47.6–2.3 (2.42–2.3)	47.5–2.1 (2.21–2.1)	45.9–2.3 (2.42–2.3)	50.0–2.8 (2.95–2.8)
Space group	*P321*	*P321*	*P321*	*P321*
Unit cell Å) *a*, *b*	106.12	106.01	106.10	106.21
Unit cell Å) *c*	55.62	55.44	60.12	60.01
No. unique reflections	16232 (2312)	19657 (2982)	15673 (2299)	9163(1420)
Multiplicity	7.5 (7.4)	7.6 (7.4)	6.4 (6.4)	5.6(6.3)
Data completeness (%)	99.6 (98.9)	92.9 (96.9)	90.0 (92.4)	92.5(100)
* R*_sym_ 1(%)	9.2 (48.8)	7.3 (49.4)	8.9 (52.0)	7.9 (49.6)
* I*/σ(*I*)	17.5 (4.8)	20.8 (4.6)	18.3 (3.9) (4.2)	21.4(4.4)
mosaicity	0.36	0.40	0.90	0.75
Refinement				
Resolution limit (Å)	15.0–2.30	15.0–2.1	15.0–2.30	15.0–2.8
Protein residues	4–237	1–244	4–237	4–237
Number of lipids	1	1	1	1
Number of water	14	26	14	14
* R*_cryst_ 2(%)	25.0	23.1	24.7	25.7
* R*_free_ (%)	27.0	25.4	26.8	27.2
Rmsd				
Bond length (Å)	0.0066	0.0056	0.0060	0.077
Bond angle (°)	1.17	1.15	1.07	1.24
B factor (Å^2^)				
Protein	39.2	29.6	32.7	50.2
water	42.7	34.0	31.8	47.7
lipids	67.4	74.5	37.9	64.8

1 *R*_sym_ = Σ_hkl_Σ_i_ |*I*_i_ - <*I*>|/Σ_hkl_Σ_i_ *I*_i_, where *I*_i_ is the intensity of an individual reflection and <I> is the mean intensity obtained from multiple observations of symmetry related reflections.

2 *R*_cryst_ = Σ_hkl_ ||*F*_obs_| - | *F*_calc_||/Σ_hkl_ |*F*_obs_|) 5% randomly omitted reflections were used for R_free_.

### The structure of the individual protein

**Figure 5:**
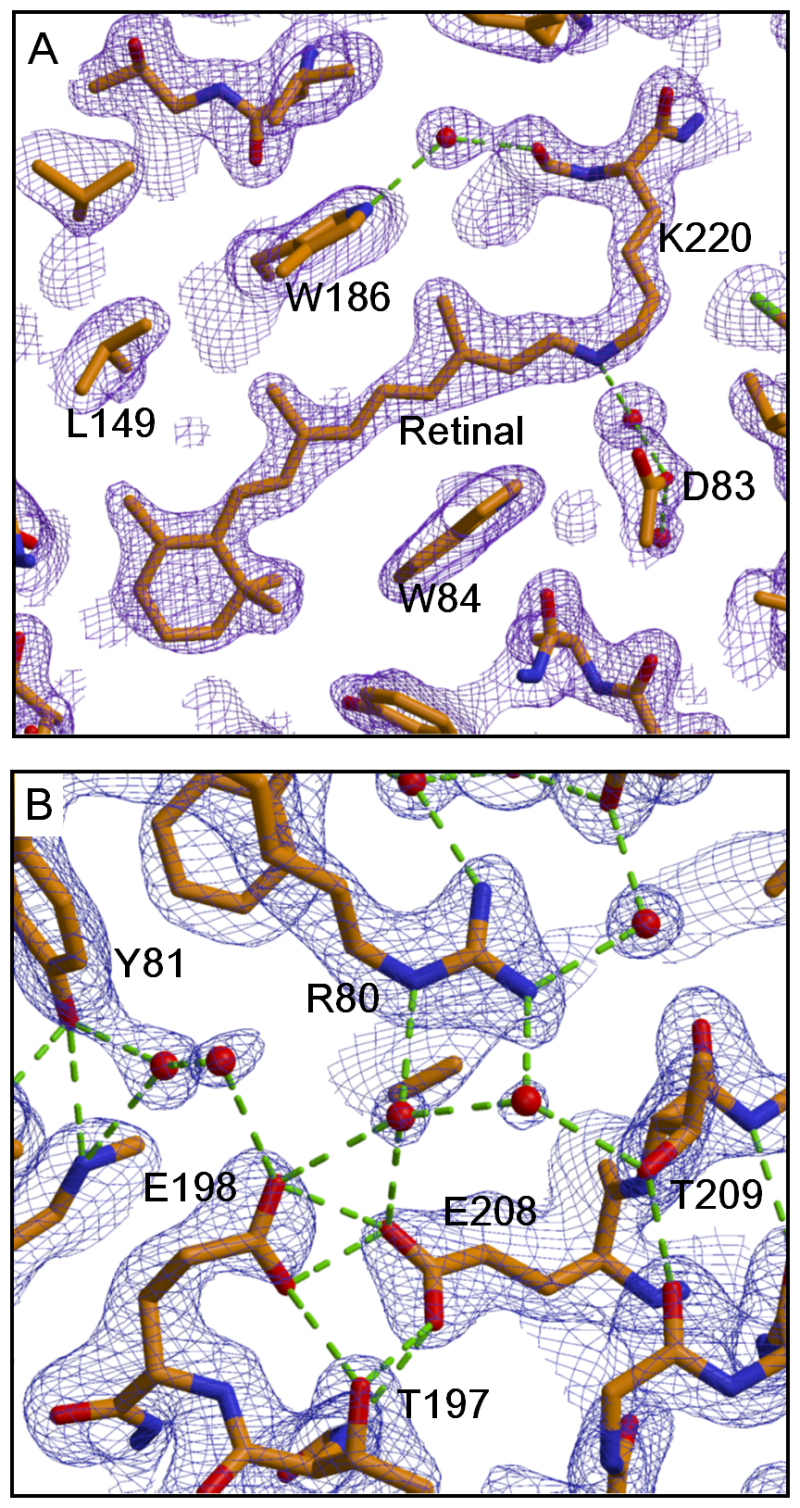
The retinal-binding pocket and the proton-release pathway in cR3. The 2*F*_o_-*F*_c_ maps around the retinal-Lys216 chain (A) and the proton-release group (B), contoured at 1 σ and overlaid on the structural model. Carbon, oxygen and nitrogen atoms are drawn in green, red and blue, respectively.

The polypeptide chain of cR3 is folded into seven trans-membrane helices (helix A through G), a β-sheet at the BC loop, and a short amphiphilic helix at the C-terminal region (Fig. 4). The retinal chromophore is bound to the ε-amino group of Lys220 in helix G and it adopts the all-trans configuration At pH 5, the β-sheet at the BC loop is tilted towards the DE loop, so that the main chain of the BC loop (at Ala70) is hydrogen-bonded to the main-chain of the DE loop (at Ala127). The C-terminal amphiphilic helix, which fills the open space between the AB and EF loops, is fixed by interactions with residues (Arg36, Arg38, Tyr41, Asp166, and Thr171) from helices B, E and F. Upon the crystal soaking at pH 6, the BC loop and the C-terminal polypeptide underwent large structural alterations and became disordered, whereas the structure of the protein inside scarcely changed (Fig. 3S). In the investigated pH range (pH 4–7), two glutamates (Glu198 and Glu208) in the proton-release channel formed a paired structure (Fig. 5B). The close distance between these residues (2.4 Å) is explainable by a low-barrier hydrogen bond [27].

The long DE loop extends towards a neighboring subunit to strengthen the trimeric structure; i.e., two residues in the DE loop interact with the main chain of the N-terminal polypeptide (at Pro2) and helix B (at Leu60 and Gly61) of the neighboring subunit (Fig. 4E). Inter-subunit hydrogen bonds are also seen on the cytoplasmic side; i.e., two residues (Asn104 and Arg105) in helix D interact with residues (Ser35, Gln38 and Lys39) in helix A of the neighboring subunit. The trimeric assembly of cR3 is further strengthened by the carotenoid bacterioruberin, which binds to the crevice between neighboring subunits (Fig. S4). One terminal end of bacterioruberin is fixed by residues from helices A and B of one subunit and helices D and E of a neighbor subunit, while the other terminal is excluded from the inter-subunit crevice. This binding mode is similar to that observed in the trimeric assembly of aR2 [10].

**Figure 6:**
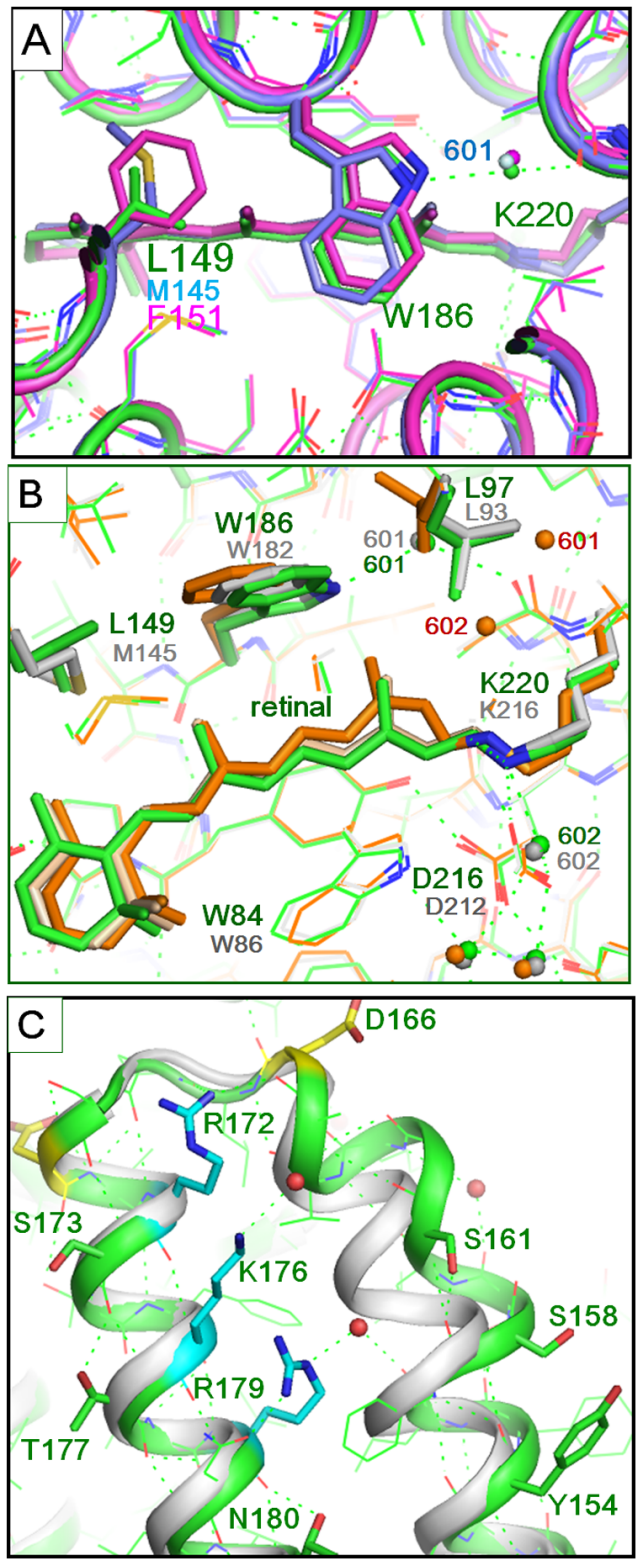
Structural comparison between cR3 and bR. A) The structure of the cytoplasmic vicinity of retinal in cR3 (green), bR (skyblue, PDB entry 1IW6) and aR2 (magenta, PDB entry 1EI4). B) The structure of cR3 (green) is compared with those of the unphotolyzed state (white, PDB entry 1IW6) and the L state (orange, PDB entry 1UCQ) of bR. C) The structure of the cytoplasmic ends of helices E and F in cR3 (green) and bR (white).

Compared with bR, peculiar features of cR3 are seen in the following regions. i) The side chain of Leu149 makes contact with the indole ring of Trp186, reducing the motional freedom of Trp186 (Fig. 6A). ii) Tyr81 OH is hydrogen-bonded to Thr124 OH, enlarging a cavity between Tyr81 and Glu198 (Fig. S5). iii) The cytoplasmic end of helix E is greatly bent, creating a large cavity near the cytoplasmic surface between helices E and F (Fig. 6C). iv) Three positive charges (Arg172, Lys176, and Arg179) are distributed along helix F (Fig. 6C). v) Ser82 OH in helix C is hydrogen-bonded to Asn54 ND2 (Fig. 1). vi) The indole nitrogen of Trp192 in helix F is hydrogen-bonded to the main chain of helix G (at Glu208) (Fig. 1).

**Figure 7:**
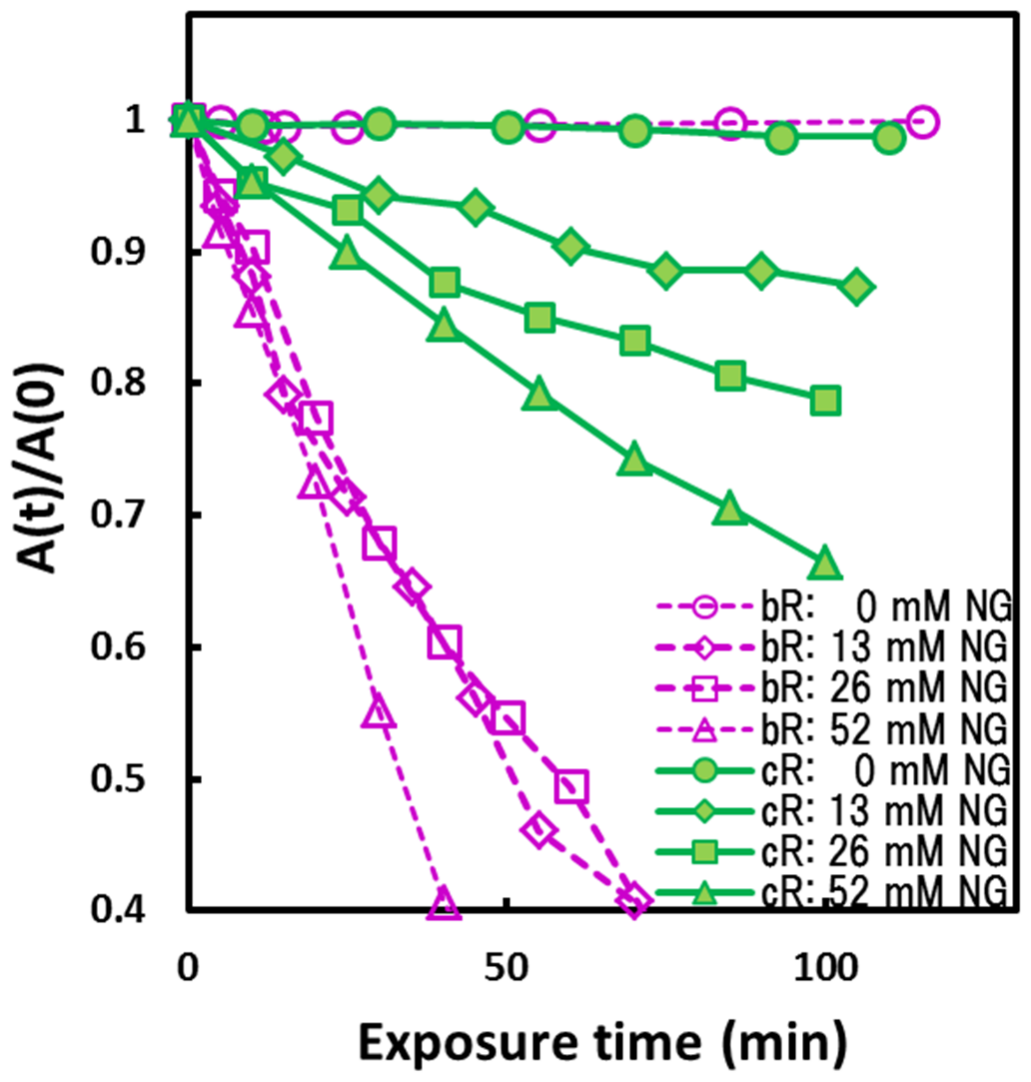
Photobleaching of the retinal chroophore in cR3 and bR at 20°C. The solubilized sample was prepared by incubating cR3-rich claret membrane or the purple membrane of *H. salinarum* in a solution containing 0.01 *M* HEPES (pH 7) and 13–52 m*M* nonylglucoside (cmc ∼6 m*M*) at 30°C for 2–24 hours); unsolubilized membranes were removed by centrifugation at 100,000 rpm. The membrane suspension or the solubilized sample was exposed to strong orange light (570–700 nm, 80 mW/cm^2^). Absorption changes at 580 nm are plotted against the exposure time. Here, A(0) is the absorbance (∼0.35) observed just after the light-adaptation (i.e., after the pre-illumination for ∼5 minutes).

A particularly interesting property of cR3 is a long DE loop that interacts with a neighboring subunit (Fig. 4E). This peculiar structure would confer cR3 with a higher ability to from a trimeric assembly. To investigate this possibility, we measured the photo-stability of cR3 in the solubilized state. When an aqueous suspension of claret membrane was exposed to strong orange light (>570 nm) from a xenon lamp, no significant absorption change was induced (Fig. 7). This result indicates that as long as cR3 is embedded in the membrane, the retinal chromophore is protected from photobleaching. Meanwhile, the light-induced bleaching of retinal became significant upon solubilization of cR3 with an excess amount of detergent (nonylglucoside). It is important to point out that the rate of this photobleaching becomes higher with the increasing detergent concentration. This dependence can be explained by supposing that the photostability of cR3 decreases when the native protein-lipid interactions and/or the trimeric structure are destroyed by an excess amount of detergent. In the case of bR, the photobleaching was already significant at a low detergent concentration (13 m*M*). At this low detergent concentration, where the photostability of cR3 was five times higher than observed for bR, a considerable fraction of cR3 seems to maintain a trimeric assembly and/or native protein-lipid interactions.

To investigate whether cR3 is able to maintain a trimeric assembly in the presence of detergent, we performed blue- native PAGE (Fig. S6). The result shows that the trimeric assembly of cR3 is maintained at a low detergent concentration (13 m*M* nonlyglucoside), whereas it is dissociated into monomers at much higher detergent concentrations.

## Discussion

### The trimeric assemblies of microbial rhodopsins

The present result shows that cR3 forms a trimeric structure with bacterioruberin bound to the crevice between neighboring subunits. Besides cR3, five microbial rhodopsins (bR, aR2, dR3, *p*HR, and halorhodopsin from *H. salinarum*) have been shown to form trimeric assemblies under crystallization conditions [5]–[7], [10], [11], [15], [16]. As long as we discuss their structures found in the 3D crystals grown by the membrane fusion method, there is no difference in the architecture of the trimeric assembly (Fig. S7). Especially the membrane-embedded region (i.e., the seven transmembrane helices) is superimposed well. [Comparison of the cR3 trimer with the aR2, bR, and dR3 trimers shows RMSD of 0.93 Å (over 618 residues), 0.98 Å (over 591 residues), and 0.56 Å (over 558 residues), respectively]. These trimeric assemblies have a common architecture: 1) helices B, C and D are aligned in parallel to the 3-fold rotation axis, whereas helices A, E, F and G are largely tilted from this axis; 2) the extracellular half of helix E makes contact with helix B of a neighboring subunit, whereas these two helices are separated on the cytoplasmic side; 3) the crevice created between neighboring subunits is filled by some lipid component. It is possible that because a flexible lipid exists in the inter-subunit crevice, the cytoplasmic half of the protein can undergo a large structural change during the proton-pumping cycle.

In the trimeric assemblies of aR2, cR3, dR3, and *p*HR, bacterioruberin was observed to bind to the inter-subunit crevice [10], [11], [16]. Nonetheless, the residues surrounding bacterioruberin are not necessarily conserved among these proteins. It appears that the binding of bacterioruberin to the inter-subunit crevice is not very specific. A recent study has shown that although the trimeric assembly of *p*HR is strengthened in the presence of bacterioruberin, *p*HR can form a trimeric assembly even in the absence of bacterioruberin [28]. We can't exclude the possibility that the cell membrane of *Haloarcula vallismortis* contains a different type of carotenoid than bacterioruberin, which binds strongly to the trimeric assembly of cR3. It has been reported that xanthorhodopsin, a light-driven proton pump found in halophilic eubacteria, utilizes salinixanthin as an antenna molecule for efficient capture of solar energy [12], [29]. It is interesting to ask whether the same energetic role is possessed by the postulated carotenoid that is bound to cR3 in *Haloarcula vallismortis*.

A recent study has shown that Gloeobacter rhodopsin, a eubacterial proton pump, forms a trimeric assembly in the presence of dodecylmaltoside [30]. It seems possible that most ion-pumping rhodopsins can form a trimeric assembly under the physiological conditions. Meanwhile, the proton-pumping activity of bR has been shown to be high even in the monomeric state [31]. An interesting question is: what merit is gained by formation of the trimeric assembly. It has been shown that the thermal stability of bR under illumination is much lower in the monomeric form than in the trimeric form [32]. One possible physiological role of the trimeric assembly is to prevent undesirable photoreactions leading to inactivation of the protein. It is noteworthy that cR3 possesses a long DE loop that interacts with a neighboring subunit (Fig. 4E). This peculiar structure would confer cR3 with a higher ability to from a trimeric assembly.

### Higher order structure of cR3

Another interesting structural feature of cR3 is the three positive charges (Arg172, Lys176, and Arg179) distributed at the cytoplasmic end of helix F (Fig. 6C). It would be expected that this cluster of positive charges affects inter-trimer interactions; i.e., cR3 trimers may not form a 2D hexagonal lattice as observed in the purple membrane of *Halobacterium salinarum*. In fact, cR3-rich claret membranes are isolated as vesicles (not planar sheets) at a low ionic strength. The cryo-electron micrographs as shown in Figure S1 suggested that cR3 trimers are arranged on a polyhedral lattice with a diameter of 50∼200 nm.

Previous studies have shown that under a special crystallization condition bR is able to form an icosahedral assembly with a diameter of 50 nm [33], and that successive fusion of such vesicular assemblies yields the *P622* crystal of bR [22]. It seems possible that the local lattice structure in the polyhedral assembly of cR3 is similar to the honeycomb lattice seen in the *P622* crystal of bR. In this case, it would be expected that cR3 trimer is arranged in such an orientation that helix F faces to a large opening space in the honeycomb lattice.

### Structural conservation of the proton-release pathway

The structure of the proton-release channel is well conserved among archaeal proton pumps. The conformations of ionizable residues (Arg86^cR3^, Asp89^cR3^, Glu198^cR3^, Glu208^cR3^ and Lys220^cR3^) in cR3 are nearly identical to those seen in bR, aR2, and dR3 (Fig. S5). A noticeable difference is seen in the orientation of the tyrosine in helix C (Tyr83^bR^, Tyr81^cR3^); i.e., Tyr81^cR3^ OH is hydrogen-bonded to the OH group of Thr124^cR3^, whereas Tyr83^bR^ OH is hydrogen-bonded to the indole nitrogen atom of Trp193^bR^. This difference is accompanied by a slight alteration in the water distribution; i..e, two water molecules are inserted between Tyr81^cR3^ and Glu198^cR3^, while only one water molecule exists between Tyr83^bR^ and Glu194^bR^.

Irrespective of these minor differences, the paired structure of two glutamates (Glu198 and Glu208 in cR3) in the proton-release channel is well conserved among aR2, bR, cR3, and dR3. It has been reported that although this paired structure is formed in the neutral purple form of bR, it is broken in an alkaline pink form (at pH>10) or an acidic blue form (at pH<3.5) [34]. In some 3D crystals (e.g., the *C2* crystal [6]) of bR, Glu194^bR^ was reported to adopt a similar conformation as observed in the alkaline purple form. It is possible that the pKa value of the alkaline transition is dependent on the protein-lipid interactions. In the *P321* crystal of cR3, the neutral purple form with the paired structure of Glu198^cR3^ and Glu208^cR3^ was stable in the investigated pH range (pH 4–7).

It has been reported that in the bR-vesicles reconstituted with egg lecithin, the neutral purple form of bR is stable in a narrow pH range; i.e., an acidic blue form with λ_max_ at 600 nm is generated below pH 4, while an alkaline red form with λ_max_ at 480 nm appears above pH 6.5 [35]. In the envelope vesicles of *Halobacterium salinarum*, on the other hand, the neutral purple form of bR exists stably in a wide pH range (pH 3–pH 9). These observations suggest that the pKa value of the purple-to-red transition is dependent on the lipid-protein interactions. This dependence should be kept in mind in the discussion of the proton-pumping activities of microbial rhodopsins expressed in neuron membranes.

### Structural factors affecting the photoreaction kinetics of archaeal proton pumps

When the proton-pumping cycle of cR3 is compared with that of bR, the most significant difference is seen in the decay rate of the K state. It is noteworthy that the kinetics of the early reaction step of the photocycle of cR3 is rather similar to that of aR2; namely, the K state decays so slowly that the L state is undetectable for kinetics reason (Fig S2). It would be expected that the decay rate of the K state correlates with the structure around the retinal chromophore. Although most residues in the retinal-binding pocket are conserved among microbial rhodopsins, Met145 in bR is replaced by leucine (Leu149) in cR3 or by phenylalanine (Phe150) in aR2. This replacement is accompanied by a noticeable shift in the position of the tryptophan residue (Trp187^aR2^, Trp186^cR3^, Trp182^bR^) that makes contact with the C13 methyl of retinal (Fig. 6A). Owing to the side chain of Leu149^cR3^ (or Phe150^aR2^), the indole ring of this tryptophan is pushed towards helix G. This implies that the motional freedom of this indole is more restricted in cR3 or aR2 than in bR. Previous crystallographic studies of the K and L states of bR have shown that the K-to-L transition is accompanied by a horizontal movement of Trp182^bR^ towards Met145^bR^ and a rotation of the side chain of Leu93^bR^ [36], [37]. It is possible that the conformational relaxation of retinal from a twisted 13-cis configuration to a planar 13-cis configuration, which takes place in the K-to-L transition, is inhibited when the structure around the C13 methyl of retinal is made rigid by the influence of the side chain of Leu149^cR3^ or Phe150^aR2^ (Fig. 6B). In cR3, the relaxation of retinal into a planar 13-cis configuration is likely to take place when the main chain of Lys220^cR^ to which retinal is covalently bound undergoes a large structural change upon formation of the L/M state [36]. A much lower decay rate of the K intermediate has been observed for proteorhodopsin [38]. Since this protein has two tryptophan residues in the cytoplasmic vicinity of retinal [14], the slow decay of its K state is accountable by the rigidity of the retinal-binding site.

Another significant difference in the photoreaction kinetics between cR3 and bR is seen in the M-to-N transition; namely, this transition occurs much faster in cR3 than in bR. This difference is attributable to a structural difference in the proton-uptake pathway. The cytoplasmic end of helix E is greatly bent so that a cavity is created between helices E and F (Fig. 6C). This cavity is large enough to accommodate a water molecule. (The number/occupancy of water molecules in this cavity may depend on the temperature.) Previous structural analyses of bR have suggested that the cytoplasmic half of helix F is tilted outwards upon formation of the N state [39]–[42]. A similar structural change has been shown to take place during the proton-pumping cycle of *p*HR in complex with azide [43]. When its long-living N/M state is generated at high pH, the cytoplasmic half of helix F is largely deformed and a linear water cluster is formed between the retinal Schiff base and Lys215^pHR^. It has been postulated that a similar linear water cluster is transiently generated during the proton-pumping cycle of any archaeal proton pump [17]. Because the unphotolyzed state of cR3 already contains a water molecule in the vicinity of Arg179 (the counterpart of Lys215^pHR^), it would be expected that the formation of a linear water cluster in the cytoplasmic inter-helical space (i.e., the formation of the N state) takes place more rapidly in cR3 than in bR. It should be pointed out that in the *P321* crystal of cR3, the M state decays very slowly (*τ*∼100 ms at 24°C). This elongated lifetime of M is explained by supposing that the opening of the cytoplasmic half is inhibited by the protein-protein interactions, as previously reported for the M-to-N transition of bR [44].

## Materials and Methods

### Expression and purification of cR3

Cruxrhodopsin-3 was expressed in a bR-deficient strain of *Halobacterium salinarum* (MPK409) according to a method used for preparation of dR3 [11]. Briefly, a *bop* gene fragment in the vector pMPK85 [45] was cloned into the vector pUC18Δ*Nde*I and, after introducing a *Nde*I site at the start codon of the *bop* gene, the 700 bp *Nde*I-*Not*I fragment was substituted with the synthetic adaptor 5′-CATATGCTCGAGGAGATCTGAGCGGCCGC. The *Bam*HI fragment in the modified vector was cloned into the *Bam*HI site of pMPK85, producing a vector pKI72. Using *Haloarcula vallismortis* genome as a template, the cruxopsin-3 (*cop3*) gene fragment was amplified using the polymerase chain reaction with the two primers CATATGGCCGCAACAGTTGGCCCA and CTCGAGTCAGGTCGGGGCAGCCGTCGG, and cloned into pKI72 at *Nde*I and *Xho*I sites. The vector with the *cop3* gene was transformed into the strain MPK409 [46]. Complete substitution of the *ura3* gene of the strain MPK409 with the *cop3* gene was achieved by positive selection with simvastatine and negative selection with 5-FOA [47].

The transformant was inoculated into a culture solution containing 0.05 m*M* uracil and, after cultivation in 20 *L* culture medium for a week at 38°C, cells were harvested and suspended in 4 *M* NaCl. Claret membrane fragments containing cruxrhodopsin-3 were isolated according to the standard procedure utilized for preparation of the purple membrane of *Halobacterium salinarum* [48].

### Preparation of bR and aR2

Purple membrane containing bR was isolated from *Halobacterium salinarum* as described previously [48]. Claret membrane containing aR2 was isolated from *Halorubrum* sp. *Aus-2* and purified according to a procedure as previously described [11].

### Blue native polyacrylamide gel electrophoresis

For estimation of the molecular weight of the lipid-protein complex in the presence of detergent, we performed blue native polyacrylamide gel electrophoresis according to the reported procure [49] with slight modifications. In this study, a low concentration of the detergent (i.e., 6 m*M* nonyglucoside) was added to an acrylamide separating gel.

### Measurement of absorption spectra and kinetics

Transient transmission data from cR3-rich claret membrane and aR2-rich claret membrane were acquired using a computer-controlled experimental setup with a digital oscilloscope and a frequency-doubled Nd-YAG laser [50]. The absorption kinetics measured at various wavelengths were analyzed using the singular value decomposition method [51].

The absorption spectrum of cR3 crystal was measured by a micro-spectrophotometer as described previously [52]. Briefly, the measuring light from Shimadzu double monochromator was passed through a pin hole with a diameter of 0.05 mm and a polarizer and focused to a single crystal of cR3 adhered to the lower glass of the crystallization kit.

### Electron microscopy

Cryogenic electron microscopy was performed as described previously [53]. Briefly, an aqueous suspension of cR3-rich claret membrane was mounted on a carbon-coated grid and, after removal of excess water, the sample was flash-cooled with liquid propane at its melting temperature. Cyo-electron micrographs were recorded with a CCD camera (Gatan SC200D) installed in a JEM2010 (Jeol) electron microscope.

### Crystallization of cR3

A high-quality crystal of cR3 was grown at pH 4 by the membrane fusion method [22]. A mixture solution containing claret membrane (∼3 mg/ml), 5 mg/ml nonylglucoside, 1 *M* ammonium sulfate, 0.08 *M* sodium chloride, 0.04 *M* sodium azide and 0.04 *M* sodium citrate (pH 4) was slowly concentrated by the sitting-drop vapor diffusion method, using 0.5 ml of 2.2–2.8 *M* ammonium sulfate and ∼0.1 *M* sodium citrate (pH 4) as a reservoir solution. Incubation at 15°C for ∼1 month yielded trigonal crystals with a typical size of 50×50×5 µm^3^. For X-ray diffraction measurements, a single crystal was picked up and soaked in a post-crystallization solution containing 2.2 *M* ammonium sulfate, 0.1 *M* pH buffer (HEPES or citrate) and 30% trehalose for ∼10 minutes; subsequently the crystal was flash-cooled in dim light with liquid propane at its melting temperature.

### Data collection, scaling and refinement

X-ray diffraction data were collected on beamline BL38B1 at SPring-8, where a crystal kept at 100 K was exposed to a monochromatic X-ray beam at a wavelength of 1.0 Å with an X-ray flux rate of ∼2×10^12^ photons/mm^2^/sec. Diffraction data were collected with an oscillation range of 1° and an X-ray flux of ∼1×10^13^ photons/mm^2^ per image. Indexing and integration of diffraction spots were carried out using *MOSFILM* 6.1 [54]. The scaling of data was accomplished using *SCALA* in the *CCP4* program suite [55]. Structural analysis was performed with *CNS* [56] and *XtalView* [57]. Firstly the structure of bR (pdb entry: 1IW6) [58] was modified using *Swiss-Model* [59], by which non-conserved residues between bR and cR3 were automatically replaced, and the whole part of the modified polypeptide was used as an input model of the molecular replacement. (The root mean square deviation between the input model and the final model of cR3 is 0.64 Å for 193 C_α_ atoms). After a rotational and translational searching with *MOLREP* [60], retinal, water and lipid molecules were added and the protein conformation was modified manually on the ground of the 2*Fo-Fc* map. Subsequent refinements including simulated annealing and individual *B*-factor refinement resulted in an *R*_cryst_ of 23.1% and an *R*_free_ of 25.4% (Table 1).

## Supporting Information

Figure S1 **Cryo-electron micrograph of cR3-rich claret membrane.**
(PDF)

Figure S2 **Flash-induced absorption changes in cR3, bR, and aR2.**
(PDF)

Figure S3 **pH dependence of the structure of cR3 in the cytoplasmic and extracellular surface regions**
(PDF)

Figure S4 **Trimeric structure of cR3 in complex with bacterioruberin.**
(PDF)

Figure S5 **The proton-release pathway in proton-pumping archaeal rhodopsins.**
(PDF)

Figure S6 **Blue-native polyacrylamide gel electrophoresis of cR3.**
(PDF)

Figure S7 **The trimeric assemblies of ion-pumping archaeal rhodopsins.**
(PDF)
