# Supplementary material for: Crystal Structure of Cruxrhodopsin-3 from Haloarcula vallismortis
Source: PLoS One. 2014 Sep 30;9(9):e108362. doi: 10.1371/journal.pone.0108362 (PMC4182453; doi:10.1371/journal.pone.0108362)
Supplement: Figure S1 — Cryo-electron micrograph of cR3-rich claret membrane. (PDF) [file pone.0108362.s001.pdf]

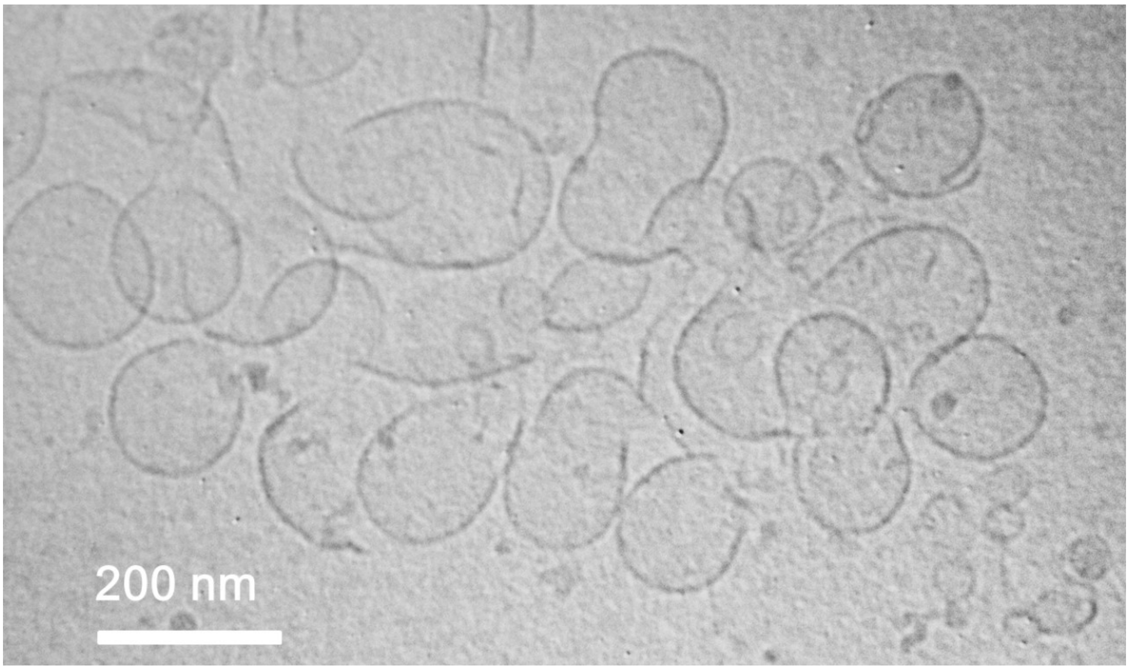

Figure S1. Cryo-electron micrograph of cR3-rich claret membrane. An aqueous suspension of cR3-rich claret membrane mounted on a carbon-coated grid was flash-cooled with liquid propane.
