# Supplementary material for: Crystal Structure of Cruxrhodopsin-3 from Haloarcula vallismortis
Source: PLoS One. 2014 Sep 30;9(9):e108362. doi: 10.1371/journal.pone.0108362 (PMC4182453; doi:10.1371/journal.pone.0108362)
Supplement: Figure S2 — Flash-induced absorption changes in cR3, bR, and aR2. (PDF) [file pone.0108362.s002.pdf]

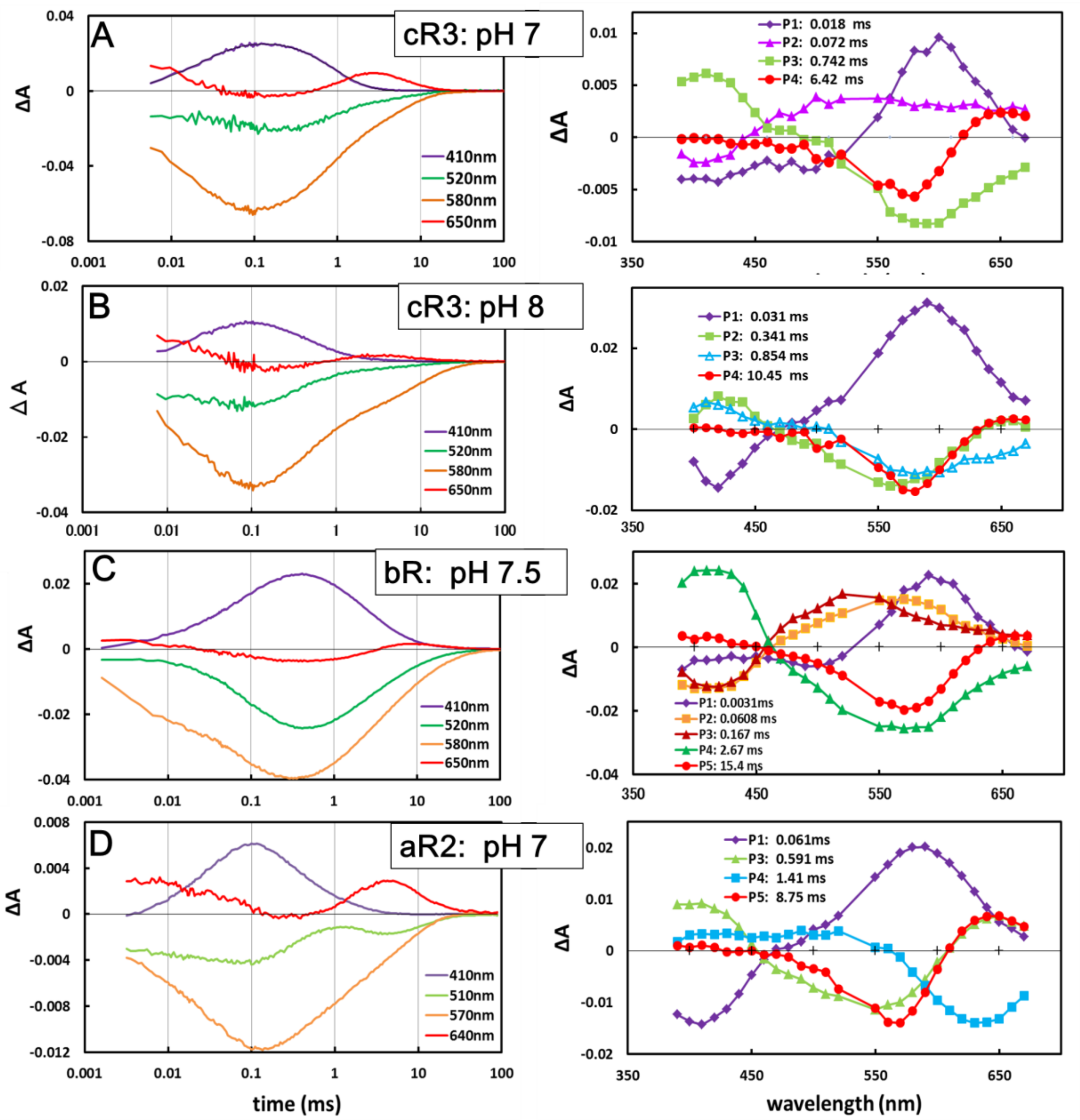

Figure S2. Photoreaction kinetics of cR3, bR and archaealrhodopsin-2 (aR2) at 24°C. A,B) Photoreaction of cR3 in the claret membrane of *Haloarcula vallismortis* at pH 7 and 8. C) Photoreaction of bR in the purple membrane of *Halobacterium salinarum* at pH 7.5. D) Photoreaction of aR2 in the claret membrane of *Halorubrum sp. Aus-2* at pH 7. Right panels: Flash-induced absorption changes observed at various wavelengths. In the investigated time range, absorption changes were fitted with four or five exponential components. Left panels: the amplitude of each exponential component is plotted against the wavelength of the measuring light.
