# Supplementary material for: Crystal Structure of Cruxrhodopsin-3 from Haloarcula vallismortis
Source: PLoS One. 2014 Sep 30;9(9):e108362. doi: 10.1371/journal.pone.0108362 (PMC4182453; doi:10.1371/journal.pone.0108362)
Supplement: Figure S3 — pH dependence of the structure of cR3 in the cytoplasmic and extracellular surface regions (PDF) [file pone.0108362.s003.pdf]

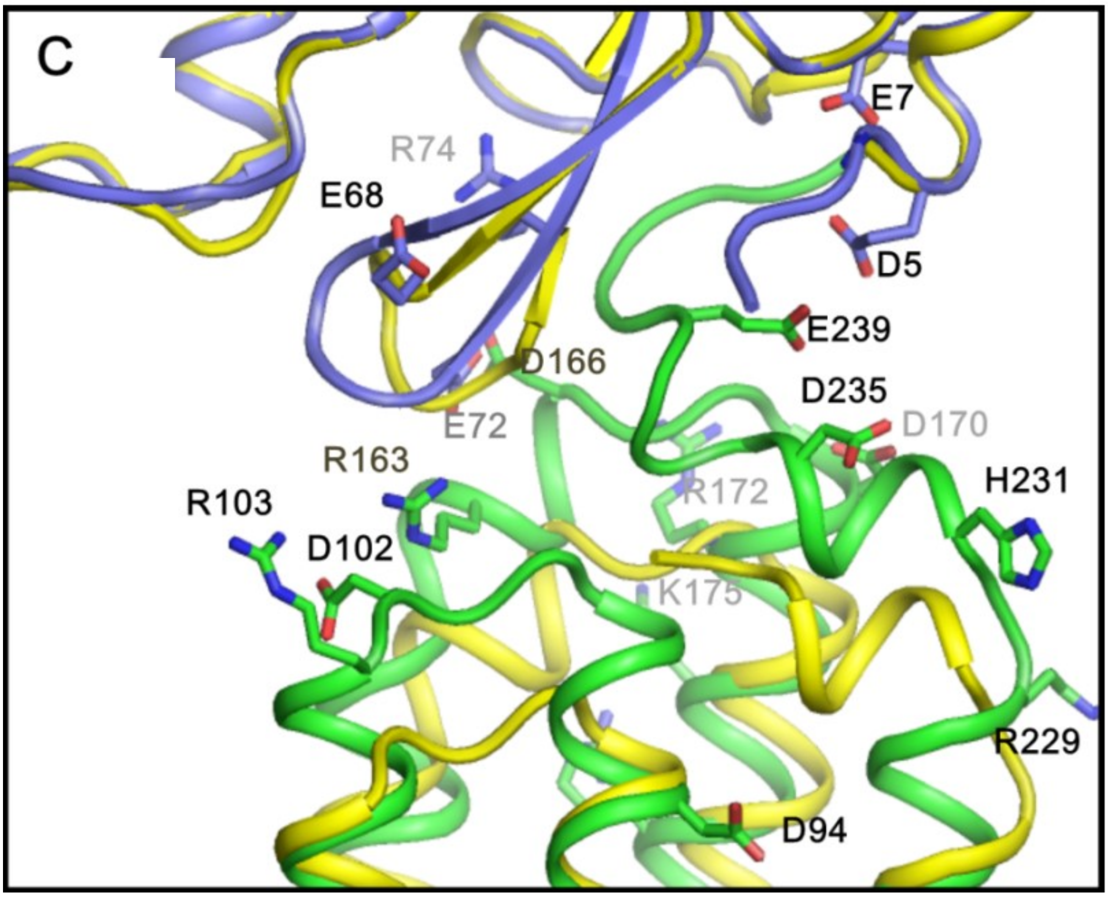

Figure S3. pH dependence of the structure of cR3 in the cytoplasmic and extracellular surface regions. The protein structure observed at pH 5 (green and blue) is compared with that observed at pH 6 (yellow). The inter-membrane space in the *P321* crystal expanded significantly in the post-crystallization soaking solution at pH 6.
