# Supplementary material for: Crystal Structure of Cruxrhodopsin-3 from Haloarcula vallismortis
Source: PLoS One. 2014 Sep 30;9(9):e108362. doi: 10.1371/journal.pone.0108362 (PMC4182453; doi:10.1371/journal.pone.0108362)
Supplement: Figure S4 — Trimeric structure of cR3 in complex with bacterioruberin. (PDF) [file pone.0108362.s004.pdf]

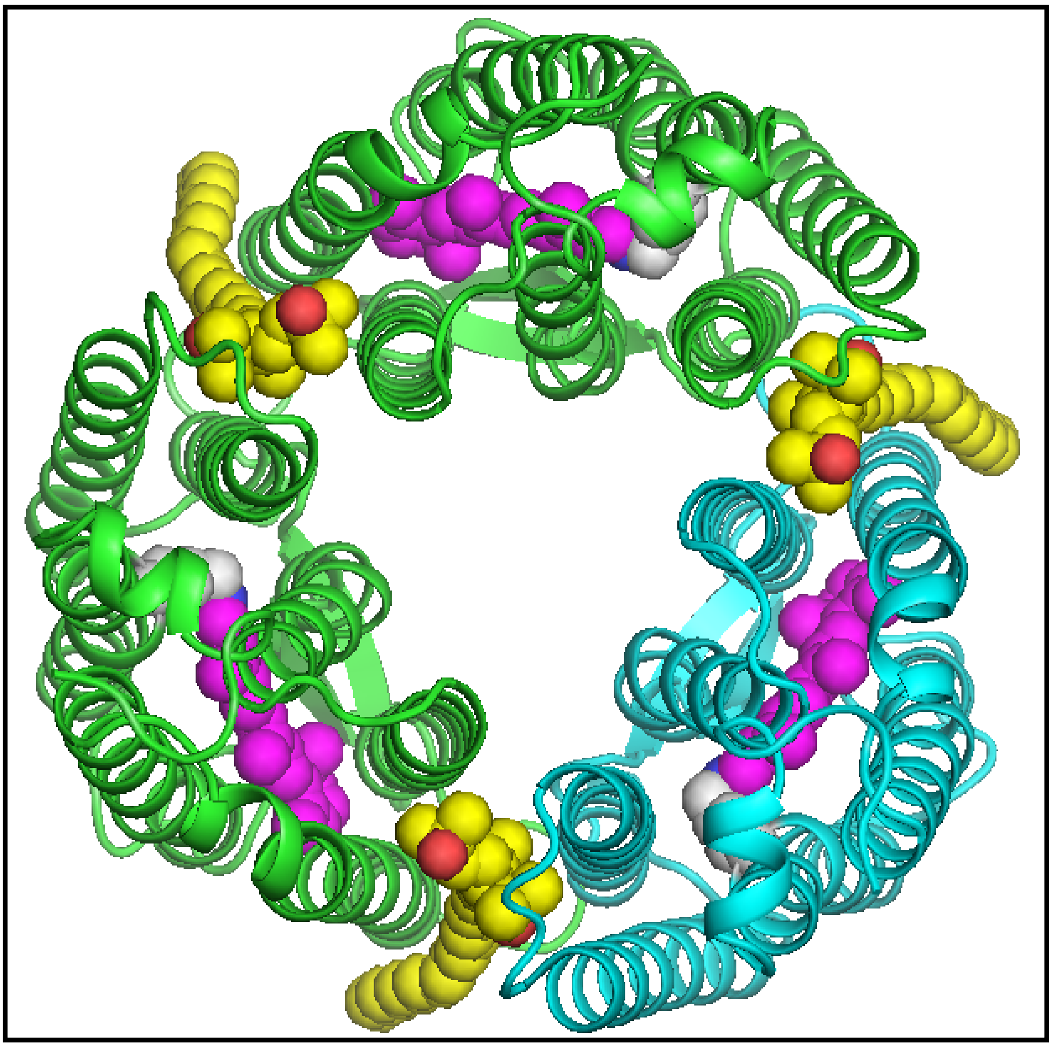

Fig. S4. Trimeric structure of cR3 in complex with bacterioruberin.
Bacterioruberin (yellow) is bound to the inter-subunit crevice within the trimeric structure of cR3.
