# Supplementary material for: Crystal Structure of Cruxrhodopsin-3 from Haloarcula vallismortis
Source: PLoS One. 2014 Sep 30;9(9):e108362. doi: 10.1371/journal.pone.0108362 (PMC4182453; doi:10.1371/journal.pone.0108362)
Supplement: Figure S5 — The proton-release pathway in proton-pumping archaeal rhodopsins. (PDF) [file pone.0108362.s005.pdf]

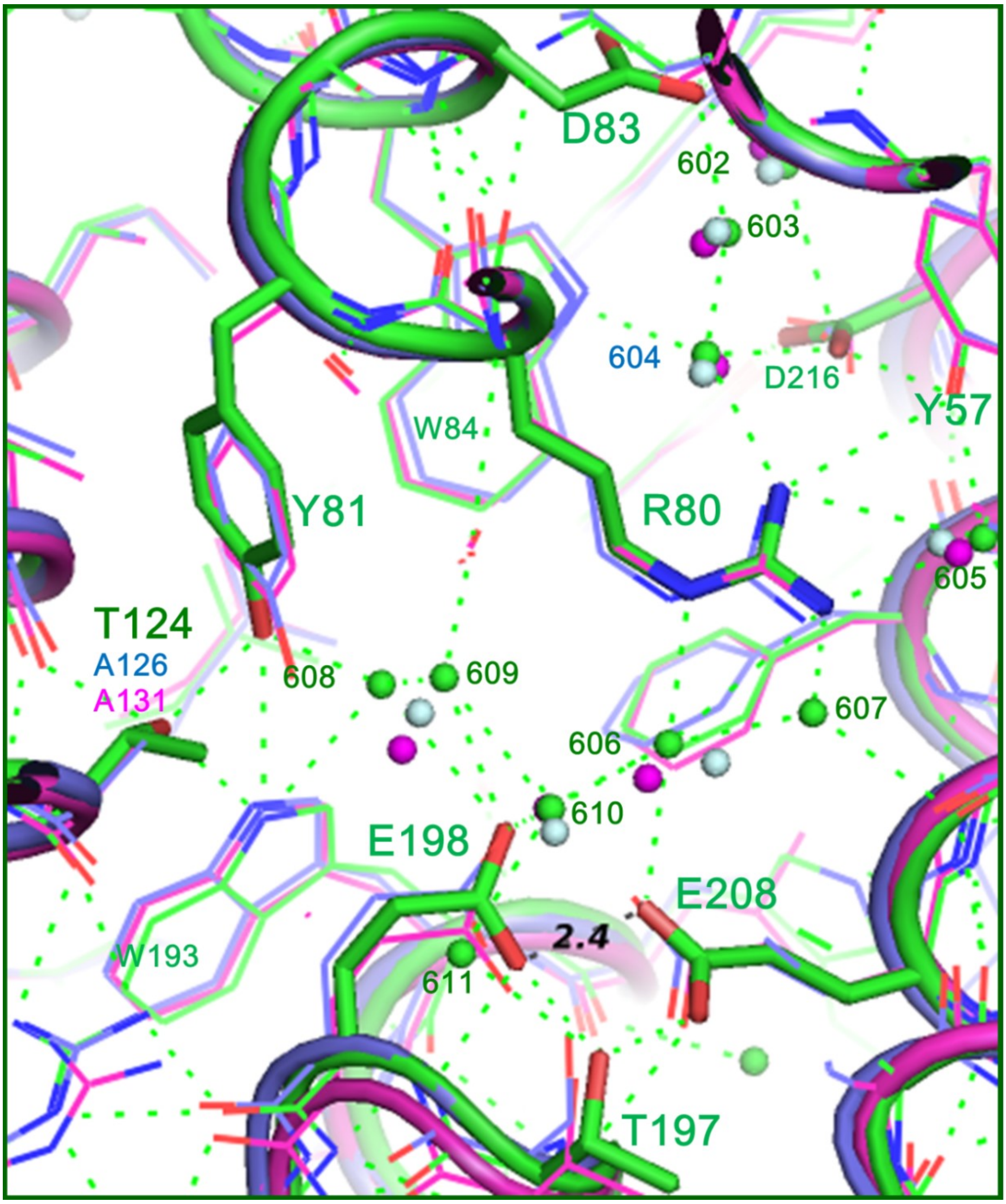

Figure S5. The proton-release pathway in proton-pumping archaeal rhodopsins. The structure of cR3 (green) is compared with those of bR in the *P622* crystal (white, PDB entry 1IW9) and aR2 in the *P321* crystal (magenta, PDB entry 2EI4).
