# Supplementary material for: Crystal Structure of Cruxrhodopsin-3 from Haloarcula vallismortis
Source: PLoS One. 2014 Sep 30;9(9):e108362. doi: 10.1371/journal.pone.0108362 (PMC4182453; doi:10.1371/journal.pone.0108362)
Supplement: Figure S6 — Blue-native polyacrylamide gel electrophoresis of cR3. (PDF) [file pone.0108362.s006.pdf]

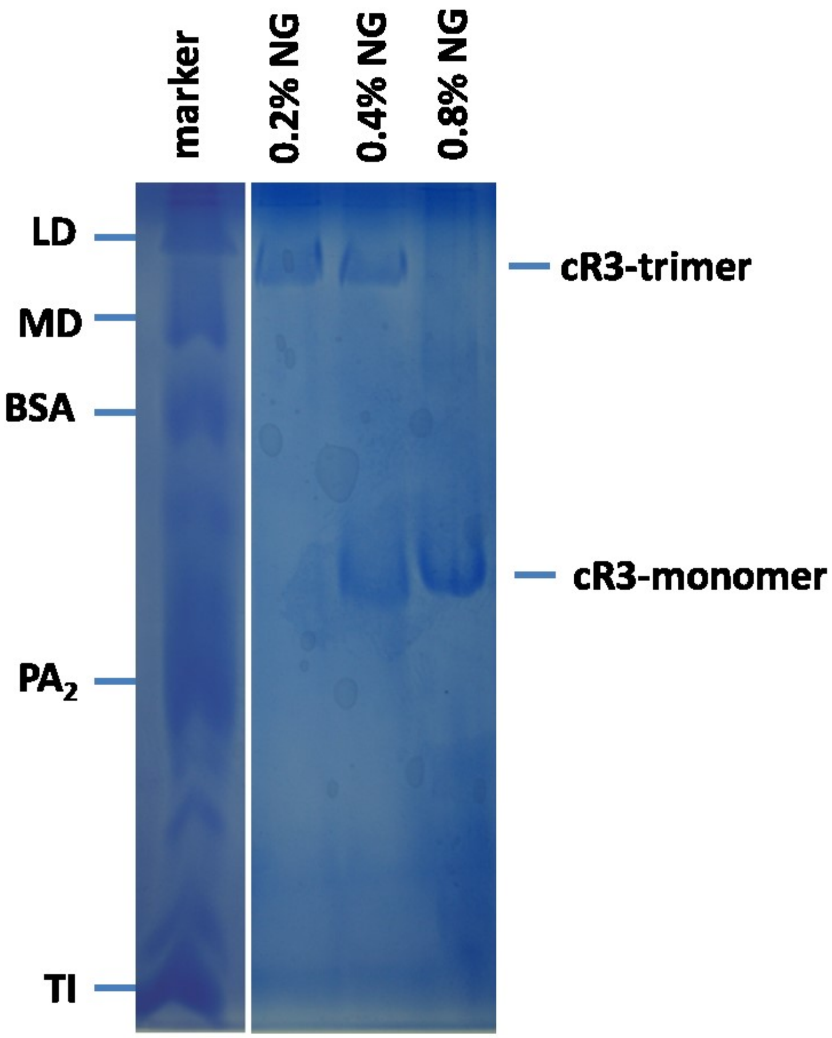

Figure S6. Blue-native polyacrylamide gel electrophoresis of cR3. cR3-rich claret membrane (~ 0.5 mg/ml cR3) was mixed with different concentrations of nonylglucoside (NG) in 0.05 *M* HEPES buffer at pH 7 and, after incubation at 30 °C for 3 hours, each solubilized sample (10 μ*L*) was mixed with 10 μ*L* of the base buffer (10 % glycerol) containing the same concentration of NG used for the membrane solubilization. Then the samples were added to the pockets of a 15% acrylamide gel containing 6 m*M* NG and overlaid with the cathode buffer containing 0.2 % Coomassie Blue G250. The electrophoresis was performed at 4 °C. The maker proteins are: LD, lactate dehydrogenase from porcine heart (tetramer: 140 kD); MD, malate dehydrogenase from porcine heart (dimer: 70 kD); BSA, bovine serum albumin (monomer: 66 kD); PLA_2, phospholipase A_2 from porcine pancrease (dimer: 26 kD); TI, trypsin inhibitor from soybean (monomer: 22 kD).
