# Supplementary material for: Crystal Structure of Cruxrhodopsin-3 from Haloarcula vallismortis
Source: PLoS One. 2014 Sep 30;9(9):e108362. doi: 10.1371/journal.pone.0108362 (PMC4182453; doi:10.1371/journal.pone.0108362)
Supplement: Figure S7 — The trimeric assemblies of ion-pumping archaeal rhodopsins. (PDF) [file pone.0108362.s007.pdf]

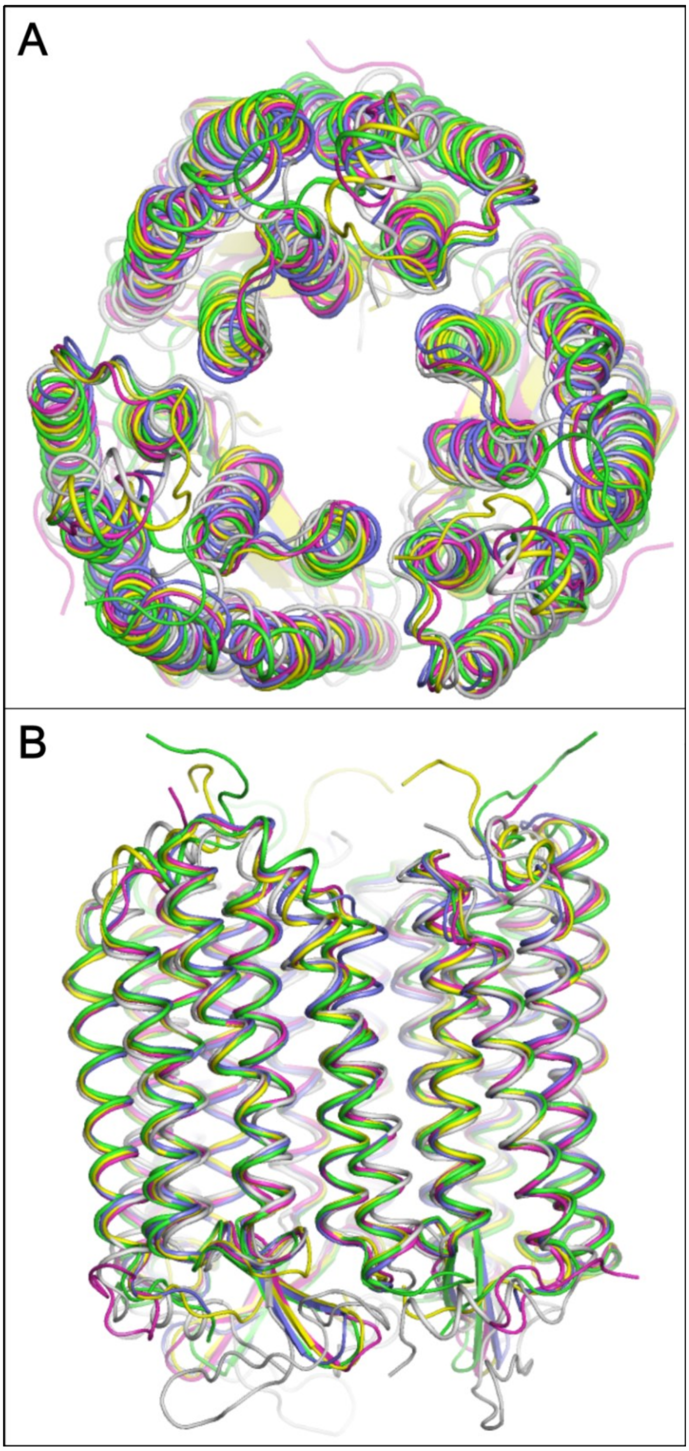

Figure S7. **Comparison of the trimeric assemblies of ion-pumping archaeal rhodopsins.** The trimeric structure of cR3 (purple, PDB entry 4L35) is compared with those of aR2 (magenta, PDB entry 2EI4), bR (orange, PDB entry: 1IW6), deltarhodopsin-3 (blue, PDB entry 1FBZ) and *p*HR (gray, PDB entry 3A7K). A) View from the cytoplasmic side. B) Side view.
